# A cost-effective strategy for identifying *Angiostrongylus* spp. larvae in *Achatina fulica*: combined morphological and molecular biology

**DOI:** 10.1186/s13071-024-06644-4

**Published:** 2025-02-09

**Authors:** Ling Jiang, Tianmei Li, Yingrui Jiang, Yuhua Liu, Shaorong Chen, Hongkun Liu, Wen Fang, Shenhua Zhao, Rong Li, Yunhai Guo

**Affiliations:** 1 Institute of Schistosomiasis Prevention and Control, Dali Prefecture, Dali, 671000 Yunnan China; 2 Chinese Center for Disease Control and Prevention, Chinese Center for Tropical Diseases Research, Key Laboratory on Parasite and Vector Biology, Ministry of Health, Shanghai, 200025 China

**Keywords:** *Angiostrongyluscantonensis*, Intermediate host, *Achatina fulica*, Morphology, Molecular biology, Combined test

## Abstract

**Background:**

The detection of *Angiostrongylus* spp. larvae in intermediate host snails is a critical epidemiological investigation, essential for the effective control of disease outbreaks. Compared to molecular biological detection methods, lung microscopy, a traditional pathogen morphological detection approach, is susceptible to oversights and exhibits relatively lower sensitivity. However, we posit that lung microscopy offers irreplaceable advantages in the context of large-scale field surveys and can serve as a vital foundation for use in conjunction with other diagnostic technologies.

**Methods:**

In this study, 348 *Achatina fulica* samples were examined using lung microscopy, PCR, and AcanITS1 qPCR. Statistical analysis was conducted to compare detection rates and sensitivities among these methods. DNA from a snail confirmed positive by lung microscopy was diluted and tested using PCR and AcanITS1 qPCR to assess the diagnostic efficacy of the molecular assays. Finally, we combined the highly sensitive AcanITS1 qPCR with lung microscopy for identifying *Angiostrongylus* spp. larvae in *Achatina fulica* for the first time to our knowledge and compared its diagnostic efficacy with that of individual testing methods.

**Results:**

The lung microscopy, PCR, AcanITS1 qPCR, and combined test yielded detection rates of 29.31%, 32.18%, 38.22%, and 38.51%, respectively. These differences were statistically significant (*X*^2^ = 9.565, *p* < 0.05). Notably, AcanITS1 qPCR demonstrated superior sensitivity with a detection threshold of 10 pg/μl, outperforming the PCR with a threshold of 10 ng/μl. When PCR was utilized as the gold standard, the sensitivities for lung microscopy, AcanITS1 qPCR, and the combined test were determined to be 88.39%, 97.32%, and 98.21%, respectively. Correspondingly, the specificities were 98.73%, 89.83%, and 89.83%, respectively.

**Conclusions:**

This novel straregy, the combined test for the detection of *Angiostrongylus* spp. larvae in *Achatina fulica* exhibited superior positive detection rates and sensitivity compared to each of the three individual methods. We believe that this novel strategy is not only applicable to large-scale field investigations of *Achatina fulica* and *Pomacea canaliculata* but also has potential application value for monitoring the infection of these snails sold at the local farmers’ markets with *Angiostrongylus* spp. larvae. Of course, while qPCR is exceptionally sensitive, the potential for false negatives remains a consideration. Repeated experimentation is also essential to maximize the reliability and accuracy of the outcomes.

**Graphical Abstract:**

**Figure:**
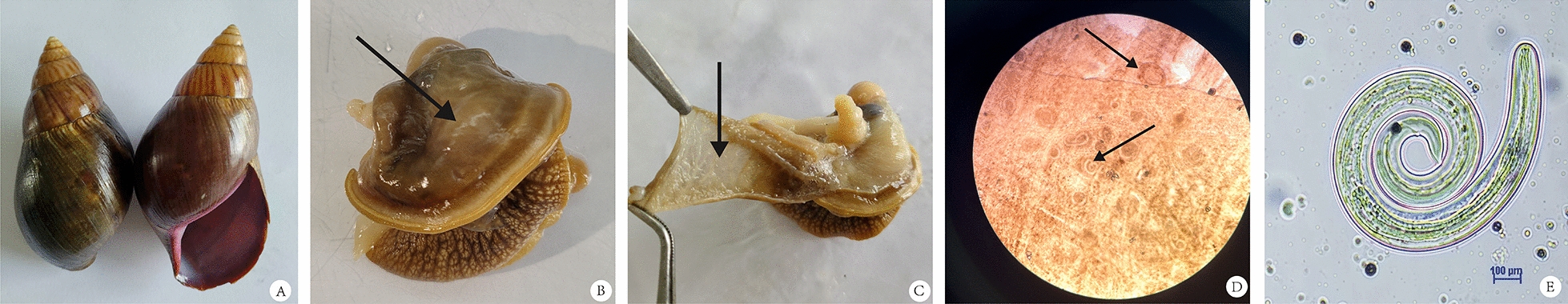

**Supplementary Information:**

The online version contains supplementary material available at 10.1186/s13071-024-06644-4.

## Background

*Angiostrongylus cantonensis* (Chen, 1935), a pivotal human parasite, is responsible for significant public health concerns, notably eosinophilic meningoencephalitis and meningoencephalitis. Infection in humans can occur through the ingestion of intermediate and paratenic hosts harboring the third-stage larvae of *A. cantonensis* or by ingesting contaminated agricultural produce, fruits, and potable water, resulting in eosinophilic meningitis [1–4]. Among them, the intermediate hosts mainly include snails such as *Achatina fulica* and *Pomacea canaliculata*, and the paratenic hosts mainly include frogs crabs, shrimps, fish, and lizards [1–4]. Angiostrongyliasis is a highly dangerous infectious disease [5], with documented cases across more than 30 countries worldwide [6], including several provinces in China [7]. *Angiostrongylus cantonensis* has also expanded its range, with worldwide distribution in Southeast Asia, East Asia, South Asia, Australia, North and South America, Europe, and Africa [8].

*Achatina fulica* Bowdich, 1822, colloquially referred to as the African giant snail and Eastern wind snail, prefers polluted environments [9]. Indigenous to East Africa, *A. fulica* is distinguished by its broad and diverse diet, resilience, and robust reproductive capabilities. Its presence not only poses a significant threat to the economic productivity and ecological balance of invaded regions but also acts as a primary vector for zoonotic parasites, such as *A. cantonensis*, thereby propagating food-borne parasitic diseases and posing a risk to public health [10–12]. *Achatina fulica* has been listed among 100 of the worst invasive species, and it has emerged as a significant invasive species in numerous countries, with widespread distribution worldwide [13, 14]. In China, *A. fulica* and *P. canaliculata* have become the two major taxa investigated as intermediate hosts of *A. cantonensis* [15], and natural infections with *A. cantonensis* intermediate hosts are present in several provinces (autonomous regions) [16], including Fujian [17], Guangdong [18], Guangxi [19], and Hainan [20]. Moreover, with the diversification of dietary preferences, certain populations prefer to consume snail meat by foraging or purchasing it at local farmers’ markets, or even eating it raw. This practice undoubtedly increases the risk of angiostrongyliasis infection among the populace [21]. Consequently, the identification of *Angiostrongylus* spp. larvae in snails has become an important epidemiological investigation, and a cost-effective, specific, and sensitive detection method has become an important condition for the effective control of angiostrongyliasis outbreaks and epidemics [22, 23].

Currently, the principal methodologies for detecting the third-stage larvae of *A. cantonensis* in intermediate host snails encompass lung microscopy, tissue homogenization, artificial digestion, and the recently introduced radula pressing for pathogenic morphological identification [23, 24]. Additionally, molecular biology techniques such as PCR, quantitative real-time PCR (qPCR), loop-mediated isothermal amplification (LAMP), and recombinase polymerase amplification (RPA) are utilized for nucleic acid detection [23, 24]. Among these, lung microscopy offers a detection efficacy comparable to tissue homogenization and artificial digestion, yet it is more straightforward to perform, requires fewer instruments and consumables, and is more expeditious, making it well suited for the qualitative screening of *Angiostrongylus* spp. larvae in large-scale field surveys of snails [25, 26]. However, the morphological detection method demands a high level of expertise and technical proficiency from the detector [27]. Factors such as the intermediate host snails being infected early or having low or moderate infection as well as the professional standard of the detectors make the detection process prone to leakage [28]. Molecular detection and amplification technologies boast high detection efficiency and sensitivity, and their operational procedures can be standardized. Nevertheless, these methods necessitate specific instrumentation and stringent operational protocols, and they incur relatively higher detection costs, making them more appropriate for laboratory research [23]. As early as 2007, a comparative analysis of three morphological detection methods (lung microscopy, tissue homogenization and artificial digestion) for identifying *Angiostrongylus* spp. larvae in intermediate host snails was conducted. Scholars have suggested that molecular detection techniques or combined applications are needed to solve the problem when numerous pathogenic snail species are to be detected [25]. Nonetheless, such studies remain scarce to date. This insight underscores the unique advantages of morphological detection methods like lung microscopy in large-scale field investigations, which, despite their limitations, offer irreplaceable benefits over molecular techniques and can serve as a crucial adjunct in combined detection strategies. In this study, we compared the efficacy of lung microscopy, PCR, and AcanITS1 qPCR in identifying *Angiostrongylus* spp. larvae in *A. fulica*. Subsequently, we innovatively combined lung microscopy with AcanITS1 qPCR, a highly sensitive molecular detection technique, in an experiment, and their respective efficacies were compared and evaluated.

## Methods

### Source of materials

**Fig. 1:**
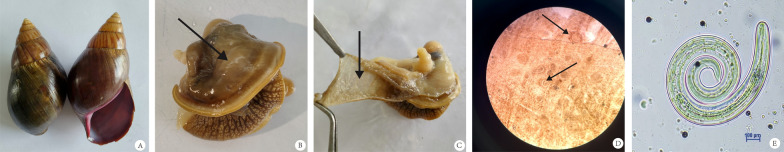
**A** Appearance and morphology of the sample of *Achatina fulica*; **B** and **C** the black arrow shows the appearance and morphology of the lung sac of *A. fulica*; **D** the two black arrows show the microscopic larval nodules within the lung sac; **E** morphology of third-stage larvae of *Angiostrongylus cantonensis*, as observed under the Nikon Eclipse ci (10×)

The samples (total 348) were sourced from the Danzhou region in Hainan and exhibited morphological traits consistent with those of *A. fulica* (Fig. 1A) [29].

### Main instruments and reagents

The primary equipment utilized in this study comprised an optical microscope, high-speed centrifuge, PCR apparatus, microwave oven, water bath, electrophoresis device, electrophoresis tank, gel imaging system, qPCR instrument, and a medical work purification table. Additionally, standard laboratory tools such as scissors, tweezers, and pipette guns were employed. The principal reagents included a DNA extraction kit, PCR and qPCR reaction pre-mix solutions, 50× TAE buffer, proteinase K, and DNA Marker, along with agarose gel powder.

### *A. cantonensis* detection methods

#### Lung microscopy

The examined snails (total 348) were dissected by removing the shell and cutting along the left side of the mantle to the posterior base, followed by the excision of the lung sac (Fig. 1B and C). The presence of any protuberant larval nodules within the lung sac was then inspected using a dissecting microscope for the preliminary screening of *A. cantonensis*. Detection of larval nodules under microscopic examination indicated a positive result for the initial screening (Fig. 1D).

Subsequently, these nodules were carefully extracted and subjected to pressing to assess the presence and morphology of the larvae under the microscope (Fig. 1E) [24, 25]. Larvae that matched the morphological characteristics of third-stage *A. cantonensis* larvae were classified as positive, while the absence of such larvae resulted in a negative outcome.

#### PCR

Template DNA from the lung sac of *A. fulica* (total 348) was meticulously extracted following the protocol of the blood/cell/tissue genomic DNA extraction kit (Tiangen, Shanghai, China) and subsequently stored at − 20 °C in individual tubes. Primers [2, 30] (Table 1) were custom-synthesized by Sangong Bioengineering (Shanghai) Co., Ltd., based on referenced sequences. The amplification reaction system for the target gene fragments was prepared with a total volume of 25.0 μl, comprising 2.0 μl of template DNA, 12.5 μl of 2 × SanTaq PCR Mix, 1.0 μl each of the forward and reverse primers (10 µM), and deionized water to reach the final volume. A negative control lacking a DNA template was included. The amplification conditions were as follows: initial denaturation at 94 °C for 5 min, followed by 35 cycles of denaturation at 94 °C for 30 s, annealing at 50 °C for 30 s, and extension at 72 °C for 60 s, with a final extension at 72 °C for 10 min and a hold at 4 °C. Post-amplification, 5 μl of the PCR product was subjected to electrophoresis on a 1.5% agarose gel, and the outcome was visualized and documented using a gel imaging system. Amplification of the anticipated band (approximately 700 bp) determined the positive and negative results. A PCR product with the expected band was forwarded to Sangon Bioengineering (Shanghai) Co., Ltd., for sequencing. The resulting sequence was deposited in GenBank to acquire an accession number, and BLAST analysis was conducted to confirm the presence of the target gene fragment.

**Table 1:** Primers or primer/probe combinations used for both molecular assays

Detection methods	Forward primer	Reverse primer	Probe
PCR	ACGTCTGGTTCAGGGTTGTT	TTAGTTTCTTTTCCTCCGCT	–
AcanITS1qPCR	TTCATGGATGGCGAACTGATAG	GCGCCCATTGAAACATTATACTT	6-carboxyfluorescein-ATCGCATATCTACTATACGCATGTGACACCTG-BHQ

#### AcanITS1 qPCR

The primer/probe sets [31] (Table 1) were custom-synthesized by Sangong Bioengineering (Shanghai) Co., Ltd. The amplification reaction mixture was formulated with a total volume of 20 μl, consisting of 10 μl of Probe qPCR Mix (2×), 0.8 μl of probe, 0.4 μl of each forward and reverse primer (10 μM), and 2 μl of DNA template, and the balance was made up with sterilized water (6.4 μl). The reaction mixture was then subjected to amplification in a qPCR instrument. The thermocycling conditions were as follows: an initial denaturation at 95 °C for 30 s, followed by 40 cycles of 95 °C for 5 s and 60 °C for 34 s. Upon completion of the amplification, samples exhibiting distinct quantitative real-time PCR amplification curves were designated as positive, with their corresponding Ct values being recorded; samples lacking these curves were classified as negative.

#### Combined test

Lung microscopy combined with AcanITS1 qPCR (in parallel): Initially, samples were assessed using lung microscopy, a morphological method for pathogen detection, to identify *Angiostrongylus* spp. larvae. Samples that tested positive for the characteristic larval morphology were recorded as such. Those deemed negative by lung microscopy were subsequently subjected to AcanITS1 qPCR analysis. The final determination of a positive result was based on the presence of distinct amplification curves; in the absence of such curves, the samples were classified as negative.

### Statistical analysis

Categorical data were expressed as *n* (%). The chi-square test was utilized to assess the comparison of detection rates across various diagnostic methods, with a significance threshold set at *α* = 0.05. A *p*-value < 0.05 was considered to indicate statistical significance. To appraise the performance of individual or combined diagnostic approaches, we calculated sensitivity, specificity, positive predictive value, negative predictive value, and the kappa statistic.

## Results

### Observation of worms in lung microscopy

Morphological characteristics of the third-stage larvae of *A. cantonensis* [24–26, 32]: under microscopic examination, these larvae are colorless and transparent, exhibiting a body sheathed in two layers, with a bluntly rounded head and an abruptly attenuated tail segment. Two chitinous rods can also be observed on the head. This is an important characteristic for differentiating the third-stage larvae of *A. cantonensis*. The dimensions of the larvae range from 462–526 µm in length and 22–27 µm in width. Internal structures such as the esophagus, intestinal tube, excretory pore, anal pore, and germinal primordium are distinctly observable. Live larvae display vigorous motility, whereas dead larvae appear dark and have indistinct outlines, with no observable peristaltic movement. The morphology of the worms observed by microscopic examination in the present study is consistent with this description (Fig. 1E).

### PCR amplification and sequence comparison

The PCR amplification yielded a 693-bp fragment of the ITS1 gene from the larvae, aligning with the anticipated size (see Additional file 1: Figure S1), and was assigned GenBank Accession no. PQ213488. BLAST analysis revealed that the sequence obtained in this study exhibited a high degree of similarity to the sequence of *A. cantonensis* (GenBank: OR790454), with 100% query cover and 98.85% percentage of similarity.

### AcanITS1 qPCR

**Fig. 2:**
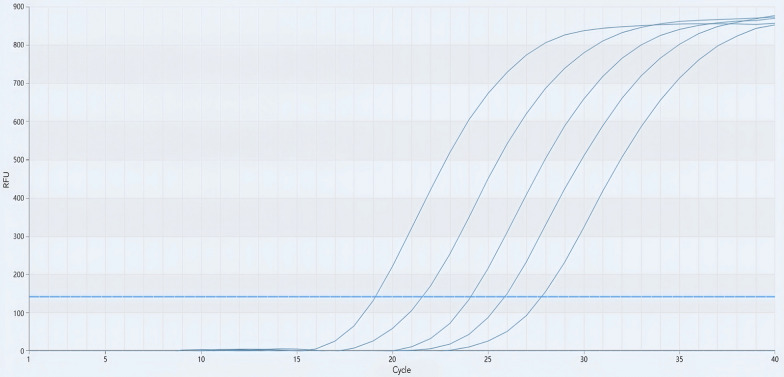
Quantitative real-time PCR amplification curves of positive samples

All positive samples displayed distinct amplification curves, with an average Ct value of 24.21. In contrast, the negative samples lacked any discernible amplification curves (Fig. 2).

### Comparison of the effect of different methods in detecting *A. cantonensis* alone or in combination

#### Comparison of positive detection rate

**Fig. 3:**
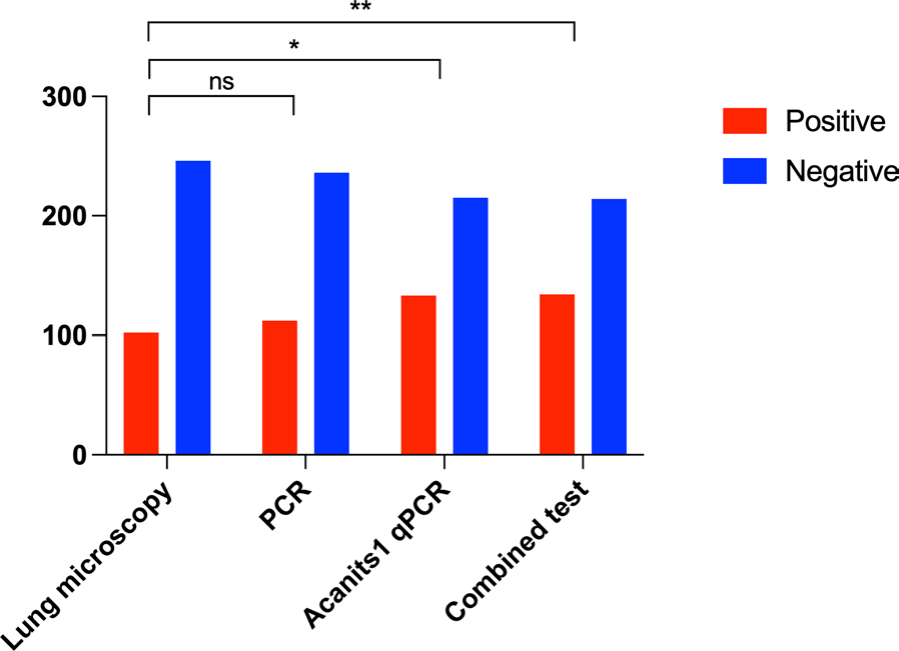
*,** The statistically significant difference (*p* < 0.05) in the detection rates between the two tests; ns, no statistically significant difference (*p* > 0.05) in the detection rates between the two tests

The detection rates of *Angiostrongylus* spp. larvae in *A. fulica*, as determined by lung microscopy, PCR, AcanITS1 qPCR, and the combined test of lung microscopy with AcanITS1 qPCR, were 29.31%, 32.18%, 38.22%, and 38.51%, respectively. The detection rates among these four tests were found to be statistically significantly different (*X*^2^ = 9.565, *p* < 0.05). Therefore, we conducted further comparative analyses to determine whether there were any statistically significant differences in detection rates between lung microscopy and other methods. Ultimately, we found significant statistical differences (*p* < 0.05) in detection rates between lung microscopy and both AcanITS1 qPCR and the combined test, whereas no statistical differences (*p* > 0.05) were observed when compared with PCR (Fig. 3).

#### Evaluation of detection efficacy

With PCR serving as the gold standard, the sensitivities of lung microscopy, AcanITS1 qPCR, and the combined test for identifying *Angiostrongylus* spp. larvae in *A. fulica* were 88.39%, 97.32%, and 98.21%, respectively. Correspondingly, the specificities were 98.73%, 89.83%, and 89.83%, respectively. The positive predictive values were 97.06%, 81.95%, and 82.09%, while the negative predictive values were 94.72%, 98.60%, and 99.07%, respectively (Table 2).

**Table 2:** Comparison of the efficacy of different methods for the detection of *Angiostrongylus cantonensis*

	PCR		Sensitivity	Specificity	Positive Predictive value	Negative Predictive value	Kappa
	+	−					
Lung microscopy							
+	99	3	88.39%	98.73%	97.06%	94.72%	0.97
−	13	233					
AcanITS1 qPCR							
+	109	24	97.32%	89.83%	81.95%	98.60%	0.94
−	3	212					
Combined test							
+	110	24	98.21%	89.83%	82.09%	99.07%	0.94
−	2	212					

+, positive; −, negative

### Comparison of the detection limits of two molecular assays for *A. cantonensis*

Template DNA from a snail confirmed to be infected with *A. cantonensis* by lung microscopy was randomly selected for analysis. This DNA was serially diluted to create six concentration gradients in a tenfold dilution series (100 ng/μl, 10 ng/μl, 1 ng/μl, 100 pg/μl, 10 pg/μl, 1 pg/μl). Subsequently, PCR and AcanITS2 qPCR were employed to amplify the template DNA at these varying concentrations. The detection thresholds for PCR and AcanITS2 qPCR were ascertained to be 10 ng/μl and 10 pg/μl, respectively (Table 3).

**Table 3:** Comparison of the detection limits of the two molecular assays

DNA concentration	PCR	AcanITS1 qPCR
100 ng/μl	+	+
10 ng/μl	+	+
1 ng/μl	−	+
100 pg/μl	−	+
10 pg/μl	−	+
1 pg/μl	−	−

+, positive; −, negative

## Discussion

At present, no definitive gold standard exists for detecting *Angiostrongylus* spp. larvae in intermediate host snails. The *Angiostrongylus* spp. larvae can be found throughout the snail's body, often forming irregular, round nodules, with a particular propensity for the lung sac, where they are more densely packed and conspicuous, thus facilitating microscopic identification [25, 33]. Consequently, for the molecular assays conducted in this study, template DNA was extracted specifically from the lung sac of *A. fulica*. We have designated PCR as the reference standard to enable a more direct visual comparison of detection efficacy among individual detection methods and between individual and combined detection methods under uniform standard conditions.

In this study, the AcanITS1 qPCR demonstrated the highest detection rate (38.22%) compared to individual detections by PCR (32.18%) and lung microscopy (29.31%). The detection sensitivity of AcanITS1 qPCR, with PCR as a control, was significantly higher than that of lung microscopy. This aligns with Qvarnstrom et al.’s [31] description of the high sensitivity of AcanITS1 qPCR and corroborates the notion that morphological detection methods like lung microscopy are more prone to underdetection compared to molecular techniques [28]. In recent years, qPCR has been widely utilized for the molecular identification and genetic diversity studies of *A. cantonensis* [2]. Its high sensitivity and practicality have rendered it applicable for the detection of *A. cantonensis* in rat peripheral blood and cerebrospinal fluid [34], and it has even contributed to the improvement of clinical diagnostics for angiostrongyliasis [35, 36]. Furthermore, researchers have employed qPCR to unravel the intricacies between mollusks and the release of parasites [37]. Regardless, these applications have substantiated the importance of qPCR in the realm of parasitic research. Additionally, the AcanITS1 qPCR exhibited a sensitivity of 97.32%, yet its specificity was 89.83%, which was somewhat lower compared to lung microscopy. This indicates that while lung microscopy might overlook some infections, it maintains a high level of specificity. However, this observation is at odds with a previous study [38], which reported that qPCR is characterized by a high degree of specificity. We attribute this discrepancy to the fact that the specificity and positive predictive values calculated in this study are relative to the gold standard, and these relative values are not equivalent to the actual values of the detection performance. Furthermore, different choices of the gold standard will yield different analysis results. Consequently, we believe that when PCR is used as a unified gold standard, the detection efficacy of the relatively insensitive lung microscopy can be well assessed, but the relative specificity and positive predictive value of the highly sensitive AcanITS1 qPCR will be underestimated. We base this viewpoint on the following reasons: first, previous studies have indicated that the sensitivity of PCR is relatively lower compared to qPCR [2]. To validate this notion, we designed an experiment in this study to determine the detection thresholds of PCR and AcanITS1 qPCR. The results showed that the detection thresholds were 10 ng/μl for PCR and 10 pg/μl for AcanITS1 qPCR, clearly demonstrating the higher sensitivity of AcanITS1 qPCR. Second, comparison of detection thresholds between PCR and AcanITS1 qPCR indicates that PCR, similar to lung microscopy, may also fail to detect early or low to moderate infections of *A. cantonensis* because of its relatively lower sensitivity. Thus, when we use the relatively insensitive PCR as the gold standard, the highly sensitive AcanITS1 qPCR correctly identifies infections that the gold standard misses. However, these correct identifications are mistakenly considered false positives by the gold standard, ultimately leading to an underestimation of the relative specificity and predictive value of AcanITS1 qPCR. Notably, we could employ the highly sensitive AcanITS1 qPCR as the gold standard to more effectively evaluate the detection efficacy of lung microscopy and PCR. However, we have compelling reasons for selecting PCR as the gold standard: first, the sensitivity of PCR lies intermediately between that of lung microscopy and AcanITS1 qPCR, allowing for a balanced assessment of the detection capabilities of both lung inspection and AcanITS1 qPCR. Second, by utilizing PCR as an independent gold standard, we can more rationally integrate lung microscopy with AcanITS1 qPCR for combined detection and directly compare the detection efficacy of the joint method with that of individual detection methods under the same criteria.

Nonetheless, it was evident that the sensitivity of AcanITS1 qPCR was markedly enhanced over lung microscopy when used in isolation. Consequently, we introduced an innovative approach that integrates lung microscopy, an essential morphological detection method, with AcanITS1 qPCR, a highly sensitive molecular technique. The combined test exhibited a detection rate of 38.51% and a sensitivity of 98.21%, which are significantly higher than the detection rate of 29.31% and sensitivity of 88.39% observed with lung microscopy. Interestingly, we also found that the efficacy of the combined detection approach was comparable to that of AcanITS1 qPCR when used alone. This suggests that in areas with high rates of natural infection in intermediate hosts, the innovative combined test can significantly enhance detection sensitivity compared to the morphological detection method of lung microscopy alone and can optimize detection efficiency. Furthermore, compared to the sole use of the molecular detection method AcanITS1 qPCR, the combined test can substantially reduce detection costs, as we strategically applied AcanITS1 qPCR only to samples initially deemed negative by lung microscopy. Consequently, we believe that the combined test also holds promise for practical application in large-scale monitoring of snails sold at local farmers’ markets for *Angiostrongylus* spp. larval infections. It is important to note that the intermediate hosts referred to here are primarily *A. fulica* and *P. canaliculata*, which possess the lung sac. Additionally, although qPCR is extremely sensitive, the possibility of false negatives cannot be ruled out. Therefore, testers must strictly pay attention to sample preservation, adhere to experimental protocols to prevent DNA degradation or cross-contamination, and repeat experiments to maximize the reliability and accuracy of the results as much as possible.

## Limitations

This study has certain limitations. *Angiostrongylus cantonensis*, initially discovered and named in Guangdong Province, China, has become the main endemic species of the Angiostrongylidae family within the country in recent years. To date, no other larvae of species within this family have been documented in China. Meanwhile, lung microscopy has proven effective in determining whether intermediate hosts are infected with* A. cantonensis*. Furthermore, numerous experiments have confirmed that feeding intermediate hosts diagnosed as positive by lung microscopy to rats (the definitive host of *A. cantonensis*) can successfully obtain the desired adults and larvae.

However, it is noteworthy that in several countries, multiple species of the Angiostrongylidae family are known to infect mollusks, which can lead to confusion and inaccurate results. Despite meticulous morphological comparisons by previous scholars between the larvae of *A. cantonensis* and other similar larvae, distinguishing the third-stage larvae of Angiostrongylidae with precision and clarity remains a formidable challenge when relying solely on microscopic examination. At this time, the microscopic data from the positive lung microscopy results were not sufficient to conclusively determine that the intermediate host was infected with the third-stage larvae of *A.* *cantonensis*; rather, the findings could only be interpreted as indicative of an infection by species within the Angiostrongylidae family. Therefore, the research methodology presented in this paper warrants further research and validation in other countries to ensure its applicability and accuracy in diverse settings.

## Conclusions

This noval strategy, the combined test, exhibited superior positive detection rates and sensitivity comapred to each of the three individual methods. We believe that thecombined test has potential application value for identifying *Angiostrongylus* spp. larvae in intermadiate host snails (*A. fulica* and *p. canaliculata*). However, the research methodology presented in this paper warrants further research and vslidation in other countries to ensure its applicability and accuracy in diverse settings

## Supplementary Information

Additional file 1. Figure S1. Gel electropherogram of PCR. Marker (M); negative samples (1–4); positive samples (5–11).

## Data Availability

No datasets were generated or analysed during the current study.

## References

[1] Hancke D, Guzman N, Tripodi M, Muschetto E, Suárez OV. Reaching new lands: updating the distribution of *Angiostrongylus cantonensis* in South America with the first record in Argentina. Zoonoses Public Health. 2024. 10.1111/zph.13163. 38937928 10.1111/zph.13163

[2] Chan AHE, Kaenkaew C, Pakdee W, Thaenkham U. Insights into the genetic diversity of *Angiostrongylus* spp. causing human angiostrongyliasis and implications for molecular identification and diagnosis. Food Waterborne Parasitol. 2024. 10.1016/j.fawpar.2024.e00230. 38827346 10.1016/j.fawpar.2024.e00230 PMC11143902

[3] Anettová L, Izquierdo-Rodriguez E, Foronda P, Baláž V, Novotný L, Modrý D. Endemic lizard *Gallotia galloti* is a paratenic host of invasive *Angiostrongylus cantonensis* in Tenerife. Spain Parasitology. 2022. 10.1017/S003118202200033. 35321776 10.1017/S0031182022000336 PMC10090600

[4] Gamiette G, Ferdinand S, Couvin D, Dard C, Talarmin A. The recent introduction of *Angiostrongylus cantonensis* and its intermediate host *Achatina fulica* into Guadeloupe detected by phylogenetic analyses. Parasit Vectors. 2023. 10.1186/s13071-023-05872-4. 37563598 10.1186/s13071-023-05872-4 PMC10416417

[5] Cheng DH, Jiang TG, Zeng WB, Li TM, Jing YD, Li ZQ, et al. Identification and coregulation pattern analysis of long noncoding RNAs in the mouse brain after *Angiostrongylus cantonensis* infection. Parasit Vectors. 2024. 10.1186/s13071-024-06278-6. 38715092 10.1186/s13071-024-06278-6 PMC11077716

[6] Huang JL, Wang Y, Zhou X. Status and control of common food-borne parasitic diseases in China: a review. Zhongguo Xue Xi Chong Bing Fang Zhi Za Zhi. 2021. 10.16250/j.32.1374.2020181. 34505454 10.16250/j.32.1374.2020181

[7] Liang MD, Lei Y, Chen K, Huang ZS, Cao XF, Yin WL, et al. Establishment of a droplet digital polymerase chain reaction method for the quantitative detection of *Angiostrongylus cantonensis* in aquatic products. J Food Saf Qual. 2023. 10.19812/j.cnki.jfsq11-5956/ts.2023.21.052.

[8] Barratt J, Chan D, Sandaradura I, Malik R, Spielman D, Lee R, et al. *Angiostrongylus cantonensis*: a review of its distribution, molecular biology and clinical significance as a human pathogen. Parasitology. 2016. 10.1017/S0031182016000652. 27225800 10.1017/S0031182016000652

[9] Chao-Qun Z, Jian-Rong D. Progress of research on biologically invasive medical molluscs in China. Zhongguo Xue Xi Chong Bing Fang Zhi Za Zhi. 2019. 10.16250/j.32.1374.2018242. 31612686 10.16250/j.32.1374.2018242

[10] Wu ZD, Huang Y, Song LG. New challenge for human parasitic disease control in China: Food-borne parasitic disease control. China Trop Med. 2019. 10.13604/j.cnki.46-1064/r.2019.01.01.

[11] Thiengo SC, Ramos-de-Souza J, Silva GM, Fernandez MA, Silva EF, Sousa AKP, et al. Parasitism of terrestrial gastropods by medically-important nematodes in Brazil. Front Vet Sci. 2022. 10.3389/fvets.2022.1023426. 36467665 10.3389/fvets.2022.1023426 PMC9715018

[12] Silva GM, Thiengo SC, Sierpe Jeraldo VL, Rego MIF, Silva ABP, Rodrigues PS, et al. The invasive giant African land snail, *Achatina fulica* (Gastropoda: Pulmonata): global geographical distribution of this species as host of nematodes of medical and veterinary importance. J Helminthol. 2022. 10.1017/S0022149X22000761. 36454026 10.1017/S0022149X22000761

[13] Lima MG, Augusto RC, Pinheiro J, Thiengo SC. Physiology and immunity of the invasive giant African snail, *Achatina* (*Lissachatina*) *fulica*, intermediate host of *Angiostrongylus cantonensis*. Dev Comp Immunol. 2020. 10.1016/j.dci.2019.103579. 31877327 10.1016/j.dci.2019.103579

[14] Celis-Ramírez M, Quintero-Angel M, Varela-M RE. Control of invasive alien species: The Giant African snail (*Lissachatina fulica*) a difficult urban public management challenge. J Environ Manage. 2022;322:116159.

[15] Guo YH, Zhang Y. Progress in the investigation of *Angiostrongylus cantonensis* intermediate host snails. J Trop Dis Parasitol. 2022. 10.3969/j.issn.1672-2302.2022.04.002.

[16] Zhang Y, Lv S, Yang K, Liu HX, Hu L, Li LS, et al. The first national survey on natural nidi of *Angiostrongylus cantonensis* in China. Zhongguo Ji Sheng Chong Xue Yu Ji Sheng Chong Bing Za Zhi. 2009;27:508–12. 20232636

[17] Xie X, Chen Y, Li Y, Xie H. Prevalence of *Angiostrongylus**cantonensis* infection in snails in Fujian Province from to. Zhongguo Xue Xi Chong Bing Fang Zhi Za Zhi. 2023. 10.16250/j.32.1374.2022197. 37455100 10.16250/j.32.1374.2022197

[18] Hu QA. The small-scale analysis of hosts of *Angiostrongylus cantonensis* and the studies on genetic diversity of *Angiostrongylus cantonensis* in Nan’ao, Guangdong Province. Chinese center disease control and prevention; 2018. https://link.cnki.net/urlid/31.1248.R.20170428.1558.042. Accessed 10 Oct 2024.

[19] Wang ZY. Investigation on the current status of Guangzhou *tubular nematode* infection in Guangxi *Fusiliers* and analysis of its genetic polymorphism. Guangxi Med Univ. 2022. 10.27038/d.cnki.ggxyu.2020.001182.

[20] Hu X, Du J, Tong C, Wang S, Liu J, Li Y, et al. Investigation on intermediate host infection of *Angiostrongylus cantonensis* in Hainan. China Trop Med. 2022. 10.13604/j.cnki.46-1064/r.2022.02.13.

[21] Huang MY, Chen DK, Chen HR, Fan MP, Liu JY, Quan YF. Investigation on *Angiostrongylus cantonensis* infection in *Achatina fulica* in Haikou wetland park. Chin J Parasitol Parasitic Dis. 2023. 10.12140/j.issn.1000-7423.2023.05.018.

[22] Wang QP, Wu ZD, Wei J, Owen RL, Lun ZR. Human *Angiostrongylus cantonensis*: an update. Eur J Clin Microbiol Infect Dis. 2012. 10.1007/s10096-011-1328-5. 21725905 10.1007/s10096-011-1328-5

[23] Zhao YB, Li TM, Guo YH. Progress of researches on techniques for detection of *Angiostrongylus cantonensis* in intermediate host snails. Chin J Schistosomiasis Control. 2023. 10.16250/j.32.1374.2022260. 10.16250/j.32.1374.2022260 37455106

[24] Zhao YB, Jiang L, Fang W, Chen SR, Liu YH, Zhao SH, et al. A new diagnostic technique for identifying *Angiostrongylus* spp. larvae in intermediate snail species by examining the buccal cavity. Parasit Vectors. 2024. 10.1186/s13071-024-06350-1. 38982497 10.1186/s13071-024-06350-1 PMC11234760

[25] Liu HX, Zhang Y, Lv S, Zhu D, Ang XH, Hu L, et al. A comparative study of three methods in detecting *Angiostrongylus cantonensis* larvae in lung tissue of *Pomacea canaliculata*. Zhongguo Ji Sheng Chong Xue Yu Ji Sheng Chong Bing Za Zhi. 2007;1:53–6. 17639702

[26] Cai WW, Lin CX, Zheng D, Xie HG. A comparative study of lung-microscopy and tissue homogenate in detecting *Angiostrongylus cantonensis* in the *Pomacea canaliculata*. China Trop Med. 2022. 10.13604/j.cnki.46-1064/r.2022.10.14.

[27] Jiang L, Zhao YB, Li TM. Species identification of the invasive *Pomacea* sp. in China. Chin J Parasitol Parasitic Dis. 2024. 10.12140/j.issn.1000-7423.2024.03.015.

[28] Wei FR, Liu HX, Lv S, Hu L, Zhang Y. Multiplex PCR assay for the detection of *Angiostrongylus cantonensis* Larvae in *Pomacea canaliculata*. Chin J Parasitol Parasitic Dis. 2010;28:355–8. 21351548

[29] Hayes KA, Cowie RH, Thiengo SC, Strong E. Comparing apples with apples:clarifying the identities of two highly invasive *Neotropical Ampullariidae (Caenogastropoda)*. Zool J Linn Soc. 2012. 10.1111/j.1096-3642.2012.00867.x.

[30] Caldeira RL, Carvalho OS, Mendonça CL, Graeff-Teixeira C, Silva MC, Ben R, et al. Molecular differentiation of *Angiostrongylus**costaricensis*, *A.**cantonensis*, and *A.**vasorum* by polymerase chain reaction-restriction fragment length polymorphism. Mem Inst Oswaldo Cruz. 2003. 10.1590/s0074-02762003000800011. 15049087 10.1590/s0074-02762003000800011

[31] Qvarnstrom Y, da Silva AC, Teem JL, Hollingsworth R, Bishop H, Graeff-Teixeira C, et al. Improved molecular detection of *Angiostrongylus cantonensis* in mollusks and other environmental samples with a species-specific internal transcribed spacer 1-based TaqMan assay. Appl Environ Microbiol. 2010. 10.1128/AEM.00546-10. 20543049 10.1128/AEM.00546-10 PMC2916500

[32] Zhang CW, Zhou XN, Lv S, Zhang Y, Liu HX. Morphology of III stage larvae of *Angiostrongylus cantonensis* in *Pomacea canaliculata*. Zhongguo Ji Sheng Chong Xue Yu Ji Sheng Chong Bing Za Zhi. 2008;26:203–9. 19160967

[33] Liu HX, Zhang Y, Lv S, Hu L, Zhou XN. Establishment and observation of the life cycle of *Angiostrongylus**cantonensis* in a laboratory setting. J Pathog Biol. 2009. 10.13350/j.cjpb.2009.11.016.

[34] Qvarnstrom Y, Xayavong M, da Silva AC, Park SY, Whelen AC, Calimlim PS, et al. Real-time polymerase chain reaction detection of *Angiostrongylus**cantonensis* DNA in cerebrospinal fluid from patients with eosinophilic meningitis. Am J Trop Med Hyg. 2016. 10.4269/ajtmh.15-0146. 26526920 10.4269/ajtmh.15-0146 PMC4710426

[35] Sears WJ, Qvarnstrom Y, Dahlstrom E, Snook K, Kaluna L, Baláž V, et al. AcanR3990 qPCR: a novel, highly sensitive, bioinformatically-informed assay to detect Angiostrongylus cantonensis infections. Clin Infect Dis. 2021. 10.1093/cid/ciaa1791. 33252651 10.1093/cid/ciaa1791 PMC8492198

[36] Jakkul W, Chaisiri K, Saralamba N, Limpanont Y, Dusitsittipon S, Charoennitiwat V, et al. Newly developed SYBR Green-based quantitative real-time PCRs revealed coinfection evidence of *Angiostrongylus**cantonensis* and *A.**malaysiensis* in Achatina fulica existing in Bangkok Metropolitan, Thailand. Food Waterborne Parasitol. 2021. 10.1016/j.fawpar.2021.e00119. 33817357 10.1016/j.fawpar.2021.e00119 PMC8005753

[37] Rollins RL, Medeiros MCI, Cowie RH. Stressed snails release *Angiostrongylus**cantonensis* (rat lungworm) larvae in their slime. One Health. 2023. 10.1016/j.onehlt.2023.100658. 38116454 10.1016/j.onehlt.2023.100658 PMC10728333

[38] Harshitha R, Arunraj DR. Real-time quantitative PCR: a tool for absolute and relative quantification. Biochem Mol Biol Educ. 2021. 10.1002/bmb.21552. 34132460 10.1002/bmb.21552

